# Determinants of multimodal fake review generation in China’s E-commerce platforms

**DOI:** 10.1038/s41598-024-59236-8

**Published:** 2024-04-12

**Authors:** Chunnian Liu, Xutao He, Lan Yi

**Affiliations:** 1 School of Public Policy and Administration, Nanchang University, Nanchang, 330031 China; 2 Digital Literacy and Skills Enhancement Research Center, Jiangxi Province Philosophy and Social Science Key Research Base, Nanchang University, Nanchang, 330031 China

**Keywords:** Information technology, Statistics

## Abstract

This paper develops a theoretical model of determinants influencing multimodal fake review generation using the theories of signaling, actor-network, motivation, and human–environment interaction hypothesis. Applying survey data from users of China’s three leading E-commerce platforms (Taobao, Jingdong, and Pinduoduo), we adopt structural equation modeling, machine learning technique, and Bayesian complex networks analysis to perform factor identification, path analysis, feature factor importance ranking, regime division, and network centrality analysis of full sample, male sample, and female sample to reach the following conclusions: (1) platforms’ multimodal recognition and governance capabilities exert significant negative moderating effects on merchants’ information behavior, while it shows no apparent moderating effect on users’ information behavior; users’ emotional venting, perceived value, reward mechanisms, and subjective norms positively influence multimodal fake review generation through perceptual behavior control; (2) feature factors of multimodal fake review generation can be divided into four regimes, i.e., regime 1 includes reward mechanisms and perceived social costs, indicating they are key feature factors of multimodal fake review generation; merchant perception impact is positioned in regime 2, signifying its pivotal role in multimodal fake review generation; regime 3 includes multimodal recognition and governance capabilities, supporting/disparaging merchants, and emotional venting; whereas user perception impact is positioned in regime 4, indicating its weaker influence on multimodal fake review generation; (3) both in full sample, male sample, and female sample, reward mechanisms play a crucial role in multimodal fake review generation; perceived value, hiring review control agency, multimodal recognition and governance capabilities exhibit a high degree of correlation; however, results of network centrality analysis also exhibit heterogeneity between male and female samples, i.e., male sample has different trends in closeness centrality values and betweenness centrality values than female sample. This indicates that determinants influencing multimodal fake review generation are complex and interconnected.

## Introduction

The rapid development of digital economy and internet has led to a growing preference for online shopping among consumers. Given virtual and uncertain nature of online shopping, consumers are increasingly relying on online reviews from early purchasers to assess product quality and merchant reputation. According to China Online Shopping Behavior Research Report (2016) published by KPMG, nearly two-thirds of consumers check others’ comments and suggestions on products when shopping online, particularly when purchasing new products^1^. Moreover, positive feedback and comments from previous consumers who have already made purchases are primary determinants influencing purchase decisions. A survey conducted by Pew Research Centre in 2016 finds that 82% of Americans would review comments before making their first online purchase^2^. Furthermore, a 2012 Nielsen survey of over 28,000 online users in 56 countries reveals that online consumer reviews are the second most trusted source of brand information, following recommendations from friends and family^3^. China is a prominent player in field of e-commerce. Taobao, Jingdong, and Pinduoduo are widely recognized as leading E-commerce platforms operating in China. Given increasing impact of online reviews on consumers’ buying decisions, shopping platforms like Taobao, Jingdong, and Pinduoduo have recognized significance of consumer reviews. Consequently, they have resorted to manipulating online reputation by adopting tactics such as posting fake reviews, thus enhancing shop rankings and brand reputation^4^. This tactic involves improving quality and quantity of reviews in an attempt to influence purchasing decisions of potential consumers, thereby resulting in a proliferation of fake reviews in e-commerce market^5^. Additionally, a study conducted by ReviewMeta finds that 16% of hotel reviews on Yelp’s website are identified as fake^6^. According to Fakespot, a fake review detection site, actual percentage of fake reviews on Amazon is estimated to be around 30%^7^. Therefore, a large number of fake reviews on e-commerce platforms may seriously affect people’s daily shopping experience.

After reviewing relative literature, we find that existing research on online fake reviews including identification of review content^8–10^, motivations behind generating fake reviews^11,12^, and impacts of fake reviews^13–15^. However, Some scholars note that current research on online fake reviews remains inadequate^16–18^. Significant gaps persist within current literature: (1) considerable attention is devoted to characterizing and detecting fake reviews, but research on underlying causal mechanisms driving fake review generation remains limited. Existing studies acknowledge influence of merchant agents, user agents, and platform agents^19,20^, but how these agents affect intrinsic mechanisms of fake review generation remains unclear; (2) prior research lacks quantitative assessments of relative importance of determinants in generating fake reviews, and fails to identify key determinants influencing fake review generation, there is also less research dividing determinants influencing fake review generation into distinct regimes. Although studies suggest that users’ purchasing preferences and merchants’ marketing strategies are determinants influencing fake review generation^21,22^, analysis of individual importance of each determinant is absent; (3) lack of research quantitatively analyzing intrinsic interrelationships among determinants of fake review generation, and oversight of interactions between these determinants. Furthermore, different influences of gender on fake review generation remain overlooked. Prior research does not explore intrinsic correlations between different motivations (determinants) of each agent, as well as heterogeneity of male and female samples within an online review context, despite evident presence of interactions among these determinants^23,24^ and different influences of gender on human behavior^25–27^.

In summary, it is necessary to further explore underlying causal mechanisms behind online multimodal fake review generation. Therefore, primary research question in this study is: how to identify causal mechanisms behind online multimodal fake review generation? This primary question encompasses three key dimensions: (1) how to find determinants that influence online multimodal fake review generation from points of platform agent, user agent, and merchant agent? (2) what are key determinants that influence online multimodal fake review generation? (3) what are inherent interconnections among determinants that influence online multimodal fake review generation and is there heterogeneity of male and female samples?

To address these questions, this study aims to explore causal mechanisms behind online multimodal fake review generation, especially: (1) to identify determinants that affect online multimodal fake review generation when platform agent, user agent, and merchant agent coexist; (2) to find key feature factor of multimodal fake review generation; (3) to explore inherent correlations among determinants that influence online multimodal fake review generation in full sample, and analyze heterogeneity of male and female samples.

In addition, our research demonstrates originality through three aspects: (1) originality lies in research question. Existing research explores determinants of fake reviewgeneration^28,29^, yet quantification of relative importance of these determinants and inherent interrelations among these determinants remains unclear. Therefore, this study focuses on finding causal mechanisms of online multimodal fake review generation. We explore identification of determinants influencing fake review generation and relative importance of each determinant. Moreover, we analyze inherent correlations between these determinants, as well as heterogeneity of male and female samples; (2) originality extends to empirical method adopted. Most of existing literature uses singular methods to conduct relative research, such as structural equation modeling^30^. This study utilizes three leading e-commerce platforms in China, namely Taobao, Jingdong, and Pinduoduo, as primary data sources. Necessary empirical data is acquired through establishment and dissemination of rigorously designed scientific questionnaires. Moreover, we investigate determinants influencing online multimodal fake review generation. By utilizing structural equation modeling, we quantify influence of these determinants on online multimodal fake review generation. Additionally, we employ machine learning technique to assess relative importance of these determinants. Furthermore, we employ Bayesian complex networks analysis to explore inherent correlations among these determinants, as well as heterogeneity of male and female samples; (3) originality lies in theoretical framework constructed. Most of existing literature employs a singular theoretical framework to analyze influence of individual interactions involving platform agents, merchant agents, and user agents on fake review generation^31^, neglecting a multifaceted theoretical approach that accounts for influence of mass interactions among different agents on fake review generation. Consequently, our study establishes a theoretical framework for online multimodal fake signal generation by integrating theories of signaling, actor-network, motivation, and human–environment interaction hypothesis. We construct a mechanistic model of online multimodal fake review generation including platform agent, user agent, and merchant agent. In addition, through employing structural equation modeling, machine learning technique, and Bayesian complex networks analysis, our findings highlight significant influence of reward mechanisms and perceived social costs. Furthermore, intrinsic correlations of these determinants, and heterogeneity of male and female samples are observed.

Compared to existing literature, this study presents an innovative approach by incorporating the theories of signaling, actor-network, motivation, and human–environment interaction hypothesis as the foundational basis. We introduce platform agent to develop an original model of online multimodal fake review generation mechanism that encompasses interactions among individuals and masses, including platform agents, merchant agents, and user agents. Our research model serves as a valuable complement to existing research models on fake review generation. Theoretical contributions of this study include three aspects: (1) prior studies primarily focus on single theories such as signaling theory and motivation theory to investigate fake review generation^32,33^, our research expands application scenarios of these four theories. By integrating the theories of signaling, actor-network, motivation, and human–environment interaction hypothesis, we construct a model for online multimodal fake review generation. Furthermore, we analyze impacts of different agents on online multimodal fake review generation. (2) existing literature on fake review generation considers influence of interactions between merchants and users on fake review generation^34^, our study broadens the scope by incorporating information interaction between individuals and mass of platforms, users, and merchants. By introducing platform agents, we enrich application of information interaction theory in study of fake reviews, and expand research scenario of fake reviews. (3) most scholars employ structural equation modeling and other individual methods to explore fake review generation^35,36^, our research integrates structural equation modeling, machine learning technique, and Bayesian complex networks analysis to explore online multimodal fake review generation mechanisms. We uncover a black box of intrinsic causal mechanisms involved in online multimodal fake review generation. We identify key determinants influencing online multimodal fake review generation and find intrinsic correlations among these determinants, as well as heterogeneity of male and female samples. Such findings contribute to a deeper understanding of online multimodal fake review generation.

## Literature review

A search of literature reveals that current research by scholars can be broadly classified into three categories.

### Determinants of fake review generation

According to previous literature, main determinants of online fake review generation can be divided into two aspects: external factors from merchants^37,38^ and internal factors from consumers^39,40^. External factors from merchants are primary driving factors of fake review generation. Fake reviews caused by external factors refer mainly to consumers making unintended comments to get rewards from merchants, as well as merchants hiring “water armies” to generate fake reviews. Nobahary et al.^41^ classify motives for posting fake reviews of online products as sales, denigration, interference, and meaninglessness. Kolhar et al.^42^ suggest that motives for posting fake reviews include merchant sales promotion, merchant rewards, consumer emotional outbursts, and malicious competition among merchants. In the field of consumer behavior, shukla et al.^43^ highlight that positive fake reviews from competitors, along with comparative advantage, serve as significant reasons for merchants to actively generate similar fake reviews. Furthermore, newly registered merchants resort to review manipulation as an initial strategy aimed at enhancing reputation. Anderson et al.^44^ note that suspected deceptive reviews are often published by real customers for non-profit purposes, such as spontaneous brand maintenance. Consumers generate fake reviews to fulfil psychological needs, such as providing help to others, enhancing social status, and communicating with others^45^. Rodríguez-Ferrándiz et al.^46^ propose that customers’ motivations for generating online fake reviews can be influenced by many factors, such as rewards, self-esteem or sense of control, and emotion venting. Wang et al.^47^ suggest that consumers may generate fake reviews due to utilitarian and hedonistic attitudes towards online review platforms. Furthermore, for firms, receiving positive reviews can boost profits and enhance reputation, giving firms strong reasons to manipulate reviews. Consumers seek financial compensation by generating negative reviews. George et al.^48^ conduct an analysis of motives behind fake reviews, identifying two aspects: one involves generating positive reviews through enhancing online influence, and the other involves hiring “water armies” to generate negative reviews.

In the field of marketing, Bianchi et al.^49^ reveal that consumers’ motivation to generate fake reviews stemmed from a desire for social benefits, financial rewards, consideration for others, and personal enhancement. Harrison-Walker et al.^50^ explore impact of incentives on consumers’ electronic word-of-mouth behavior and determine that hiring reviewers is primary factor behind fake review proliferation. Additionally, Akhtar et al.^51^ conclude that consumers engaged in generating fake reviews due to their need for social interactions, financial incentives, and self-worth reinforcement. Moon et al.^52^ identify a sense of belonging and satisfaction derived from helping others as primary motives driving fake reviews generation. Zhang et al.^53^ argue that fake reviews serve three purposes: generating publicity, offsetting poor quality with positive reviews, and disparagement. Khan et al.^54^ suggest that merchants generate fake reviews to get illegal interests, encourage purchase of poor-quality products, or prevent consumers from buying high-quality products. Mohawesh et al.^55^ categorize fake reviews based on motives such as promoting sales or seeking revenge against competitors at merchant level, and expressing dissatisfaction or seeking rewards at consumer level. Rasappan et al.^56^ suggest that presence of fake characteristics in product reviews can be attributed to various factors, including adoption of a “positive review” strategy. Additionally, implementation of a “bad review threat” strategy drives users to remove negative reviews. Moreover, review mechanisms on Taobao such as restrictions on reviewing returned products, contribute to deviation of product reviews from reality. Su et al.^57^ propose that association between fake reviews and product quality can help to distinguish underlying motivations behind fake reviews.

### Fake review identification

Research on identifying fake reviews primarily focuses on fake review generator identification^58,59^ and fake review content identification^60,61^. Mewada et al.^62^ propose a method to identify fake reviews publishers by differentiating emotional content of their reviews from that of genuine reviewers. Xu et al.^63^ develop an evidence theory model based on fusion of user behavioral evidence, achieving an 87% accuracy rate in identifying fake reviewers. Srisaila et al.^64^ propose a novel framework for PU learning, aimed at detecting fake reviews, and experimental results substantiate effectiveness of proposed method in detecting fake reviews. Cheng et al.^65^ propose a method that integrates lexical and syntactic features for identifying fake reviews, this method achieves satisfactory recognition results. Wang et al.^66^ introduce a technique based on a topic-opposite sentiment dependency model to detect fake reviews. Abrar et al.^67^ employ various machine learning algorithms to identify fake reviews. Hajek et al.^68^ construct a multi-domain golden dataset encompassing reviews from hotels, restaurants, and other domains. Duma et al.^69^ define and extract context-independent grammar rule features from reviews, employing SVM classifiers to detect fake reviews. Vidanagama et al.^70^ employ rules to filter spam reviews based on inherent characteristics of blog platforms and employ LDA to extract topics from blogs to identify spam reviews within blogosphere.

When it comes to identifying fake reviews through review content, Zhou et al.^71^ design an online deception recognition system that incorporates different deception linguistic cues and features. Zhai et al.^72^ generate psycholinguistic features of online merchandise reviews and combined them with textual features, using a support vector machine classifier for automatic classification of deceptive online reviews. Deshai et al.^73^ propose an expert identification method for processing negative word-of-mouth online. Martinez-Torres et al.^74^ use a portrait alignment compatibility model to identify fake hotel reviews by identifying clustering anomalies in reviews. Qu et al.^75^ propose a Bayesian identification model to detect fake reviews. Bathla et al.^76^ empirically modelled behavior of fake reviews by considering users’ rating behavior. By identifying origins of fake reviews to detect fake reviews. Fang et al.^77^ conduct research involving 1,470 reviews from Amazon website. By employing a single- index selection method and a five-index integration selection method to identify spam reviews. Kaliyar et al.^78^ focus on defining both intrinsic features of reviews and correlation features among reviewers based on review behavior. By employing an improved clustering algorithm to group review data and subsequently calculating deviation degree of each cluster from overall review dataset. Additionally, by utilizing clustering method, fake reviews can be identified. Ben Jabeur et al.^79^ reveal that fake reviews exhibit remarkable consistency in structure, suggesting a template-driven generation process. Building upon this work, Plotkina et al.^80^ introduce emotional features into analysis of reviews and employ a polygraph model to detect fake reviews. Le et al.^81^ expand on prior research and develop a sophisticated multidimensional time series model for detecting fake reviews, further advancing field of review authenticity assessment.

### Impact of fake reviews

Fake reviews of online products can significantly impact consumers’ willingness to purchase products. Song et al.^82^ find that maliciously negative reviews have significant effects on consumers’ perceived trust and purchase intention. Duan et al.^83^ demonstrate that online merchandise reviews have limited persuasive effects on movie consumers, but significant cognitive effects. Berger et al.^84^ investigate effects of negative reviews on product sales, noting that these effects vary between well-known and unknown brands. Costa et al.^85^ highlight potential impact of fake reviews on shaping consumer beliefs and attitudes during early and mainstream stages of product adoption. Hakami et al.^86^ emphasize detrimental effects of fake reviews on review credibility, helpfulness, and overall value. Tufail et al.^87^ point out that fake reviews can lead to feelings of dissatisfaction, deception, and distrust among consumers, influencing negative word-of-mouth and repeat purchase intentions. Paul et al.^88^ conclude that manipulated online reviews have less influence on consumer purchase decisions and firm revenues when compared to authentic reviews. Furthermore, Ansari et al.^89^ note that fake reviews may temporarily boost a firm’s visibility, increasing average ratings and review numbers. Wu et al.^90^ argue that engagement in fake reviews beyond critical threshold could lead to decreased product sales and financial gains. Barbado et al.^91^ highlight adverse effects of fake reviews on platforms, impacting consumer evaluations and trust. Wang et al.^92^ demonstrate that fake reviews exhibit high emotional polarity and readability, suggesting that consumers can be influenced by writing style of reviews, thereby influencing decision-making. Di et al.^93^ show that fake reviews heighten consumer uncertainty, diminish trust, and impact decision-making processes. Yuan et al.^94^ find that consumers are less affected by fake positive reviews. Koukova et al.^95^ conduct a study revealing that fake reviews increase buyer time costs and diminish trust in both reviews and respective stores. Additionally, in a scenario simulation experiment, Shen et al.^96^ discover that presence of bad reviews exerts a notable influence on consumers’ perceived trust and purchase intention. Petratos et al.^97^ demonstrate that online product reviews possess limited persuasive effects on movie consumers, and online product reviews do significantly impact consumers’ cognitive evaluation. Mardumyan et al.^98^ conduct a setup scenario experiment to investigate effects of negative reviews on product sales and reveal that impact of negative reviews vary depending on brand’s level of recognition, as well as specific product being evaluated.

As can be seen from above, current research on fake reviews is still limited, with more focus on determinants, identification, and impact of fake reviews, while little attention is paid to inherent causal mechanisms of fake review generation. In particular, there is a lack of: (1) attention to determinants influencing online fake review generation in coexistence of platforms, merchants, and users; (2) quantitative analysis of relative importance of determinants affecting online fake review generation; (3) quantitative research on intrinsic relationships between determinants affecting online fake review generation, as well as impact of different gender groups on online fake review generation. Therefore, by referring to scholars’ research on fake reviews, based on the theories of signaling, actor-network, motivation, and human–environment interaction hypothesis, we develop an online multimodal fake review generation model, our model encompasses platforms, users, and merchants. Additionally, we design questionnaires that corresponded to variables in model to collect data. Questionnaires are distributed electronically to users of China’s three leading E-commerce platforms (Taobao, Jingdong, and Pinduoduo). Utilizing structural equation modeling, we investigate determinants influencing online multimodal fake review generation and their impact pathways. Furthermore, we employ machine learning technique to quantify importance of feature factors and divide them into distinct regimes. Moreover, by using Bayesian complex networks analysis, we examine intrinsic correlations of these feature factors in full sample and analyze heterogeneity of male and female samples.

### Theoretical framework

We build our theoretical framework on information science theories including the theories of signaling, actor-network, motivation, and human–environment interaction hypothesis.

Signaling theory comprises three crucial components: sender, signal, and receiver^99^. Scholars have utilized signaling theory to investigate impact of online reviews as product signals on consumers’ purchasing choices. For instance, Wang et al.^100^ examine influence of review potency and quantity on consumers’ purchasing decisions. Another study conducted by Chen et al.^101^ explores effects of review potency and quantity on product sales. Furthermore, Liao et al.^102^ investigate impact of review potency on video game sales. Additionally, some research divides signals into product and seller signals^103^, while other research further subdivides signals into individuals, products, and organizations^104^. Based on signal agents emitting them, some scholars adopt a broad classification scheme differentiating between internal and external signals^105,106^. Moreover, based on signaling theory, some scholars employ agent simulation^107^ and computational experiments^108^ to simulate impact of e-commerce platform signals on dynamics of consumer and merchant review behavior. Therefore, this paper aims to explore impact of merchant agent behavioral signals, user agent behavioral signals, and platform agent signal governance on online multimodal fake review generation by utilizing signaling theory.

Actor-network theory (ANT) is not only a theoretical framework but also a research methodology, and ANT focuses on heterogeneous networks^109^. ANT emphasizes importance of heterogeneous networks in understanding social phenomena. When applying this theory, major work is to explain actor interactions within heterogeneous networks in specific contexts^110^. Versatility of actor-network theory allows for its application to nearly any context, offering a unique perspective and methodology to study constantly evolving and changing information activities. ANT is a social science research method that employs a network system model to elucidate behavioral relationships among actors. In understanding phenomenon of fake review generation, ANT offers a suitable framework and valuable perspective. Therefore, this paper examines influence of signal governance by platform actors, as well as signal-generating behaviors of users and merchants, on online multimodal fake review generation from a comprehensive perspective.

As an internal driving force, motivation plays a significant role in shaping individual behavioral intentions. Self-determination theory (SDT) posits that both extrinsic and intrinsic motivations can influence individual behavior^111^. External determinants or demands, such as goal orientation, value perception, and material rewards may trigger extrinsic motivation^112^. Intrinsic motivation stems from internal needs, it includes three fundamental psychological needs: autonomy, competence, and relatedness^113^. Although two motivations are different, they interact with each other in specific contexts. Garnefeld et al.^114^ conduct a study to examine influence of incentives on consumers’ electronic word-of-mouth (eWOM) communication behavior. They find that hiring reviewers is primary motivator for encouraging eWOM. Wu^115^ investigates determinants that motivate consumers to provide additional reviews on online platforms and proposes a new framework to explain motivation behind consumers’ additional review behavior. Therefore, this paper highlights significance of users’ and merchants’ motivation signals in generating multimodal fake reviews.

Interaction theory^116^ posits that individual behavior is determined by interaction between information environment stimulus and individual intrinsic traits. An individual’s behavior is outcome of a continuous interplay between an individual’s intrinsic traits and information environment stimuli they encounter. There exists a profound intrinsic association between individual and information environment stimuli^117^. Interplay between intrinsic traits of platforms, users, merchants and online environments determines online multimodal fake review generation. Consequently, this paper examines impact of information interactions between behavioral signals of user and merchant agents, as well as signal governance of platform agents on online multimodal fake review generation.

**Figure 1:**
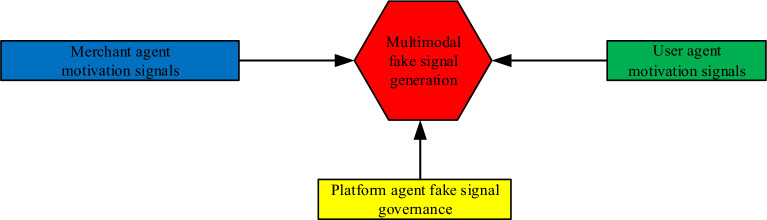
Conceptual framework of multi-agent fake signal generation. *Note*: red represents multimodal fake signal generation, blue represents merchant agent motivation signals, green represents user agent motivation signals, and yellow represents platform agent fake signal governance.

Therefore, based on the theories of signaling, actor-network, motivation and human–environment interaction, Fig. 1 presents our conceptual framework of multi-agent fake signal generation.

Based on conceptual framework, in this study, we discuss effects of emotional venting, perceived value, reward mechanisms, subjective norms, perceptual behavior control, hiring review control agency, supporting/disparaging merchants, recommending/disparaging products, perceived social costs, perceived psychological benefits, user perception impact, merchant perception impact, multimodal recognition and governance capabilities on multimodal fake review generation.

### Emotional venting, perceived value, reward mechanisms, subjective norms, and perceptual behavior control

Perceptual behavior control refers to an individual’s perception of ease or difficulty in adopting a behavior^118^. Personal traits such as resources, cognition, and expertise can influence this perception. Emotions experienced in a given situation may influence an individual’s behavior^119^. Perceived value has a positive influence on behavioral control^120^. Material rewards can stimulate users to modify their previous negative reviews and generate fake reviews^121^. Additionally, subjective norms can affect perceptual behavior control and fake review generation^122^. Based on these reports, we hypothesize that: *emotional venting significantly and positively affects perceptual behavior control* (Hypothesis 1a); *perceived value significantly and positively influences perceptual behavior control* (Hypothesis 1b); *reward mechanisms significantly and positively influence perceptual behavior control* (Hypothesis 1c); *subjective norms significantly and positively influence perceptual behavior control* (Hypothesis 1d).

### The mediating role of perceptual behavior control

Non-volitional determinants, such as necessary opportunities and resources, can influence information review behavior of individuals. Users’ perceptual behavior control plays a significant role in shaping their interaction with an online review. Increased support for a specific behavior enhances self-confidence in individuals, thereby leading to a subsequent increase in perceptual behavior control^123^. Similarly, reviews’ information will draw attention of individuals toward a product, thus impacting their perceptual behavior control^124^. Consequently, this research posits that users’ perceptual behavior control could potentially contribute to online multimodal fake review generation. Based on these reports, we hypothesize that: *perceptual behavior control significantly and positively influences multimodal fake review generation* (Hypothesis 2); *perceptual behavior control mediates relationship between emotional venting and multimodal fake review generation* (Hypothesis 3a); *perceptual behavior control mediates relationship between perceived value and multimodal fake review generation* (Hypothesis 3b); *perceptual behavior control mediates relationship between reward mechanisms and multimodal fake review generation* (Hypothesis 3c); *perceptual behavior control mediates relationship between subjective norms and multimodal fake review generation* (Hypothesis 3d).

### Hiring review control agency, supporting/disparaging merchants, recommending/disparaging products, and multimodal fake review generation

Manipulation of reviews by merchants through hiring review control agencies is a widely observed and growing phenomenon. Such reviews often exhibit an excessive bias toward product promotion or denigration^125^. Merchants hire these review control agencies for two main purposes: first, to enhance visibility of their products and services; and second, to denigrate and undermine their competitors. Hence, primary purpose of hiring a review control agency is to achieve profitability. Hiring “water armies” is an extremely effective tactic for boosting commodity sales volume^126^. Additionally, for their vested interests, some merchants hire a review control agency to generate an online review. Based on these reports, we hypothesize that: *hiring review control agency significantly and positively affects supporting/disparaging merchants* (Hypothesis 4a); *hiring review control agency significantly and positively affects recommending/disparaging products* (Hypothesis 4b); *hiring review control agency significantly and positively affects multimodal fake review generation* (Hypothesis 4c).

### The mediating role of supporting/disparaging merchants

With the rapid development of internet, competition among merchants has intensified. In context of heterogeneous multi-agent information interaction, facilitating merchants’ online reviews can effectively enhance their competitiveness. However, some merchants manipulate online reviews to misrepresent actual quality of their products^127^. When users find that a merchant fails to meet certain requirements, they will leave negative reviews of product. Moreover, Users can provide positive reviews to support merchants they approve of, thus generating multimodal fake review^128^. Users’ support or disparagement of merchants can significantly influence multimodal fake review generation^129^. Based on these reports, we hypothesize that: *supporting/disparaging merchants significantly and positively affects multimodal fake review generation* (Hypothesis 5); *supporting/disparaging merchants mediates relationship between hiring review control agency and multimodal fake review generation* (Hypothesis 6).

### The mediating role of recommending/disparaging products

To express their contentment/discontentment with shopping experiences and products, users often generate excessively positive/negative online reviews. Primary motivation for improper review is to share their specific encounter with product and service, thereby providing subsequent users with valuable insights into purchasing process^130^. Online reputation has a facilitating effect on review behavior^131^. Contextual determinants significantly impact users’ inclination to generate online reviews. One of reasons behind users’ reviewing is to support merchants’ products. Based on these reports, we hypothesize that: *recommending/disparaging products significantly and positively affects multimodal fake review generation* (Hypothesis 7); *recommending/disparaging products mediates relationship between hiring review control agency and multimodal fake review generation* (Hypothesis 8).

### Moderating effects of perceived social costs and perceived psychological benefits

Social exchange theory posits that individuals’ decision to exchange resources with others depends on their evaluation of perceived benefits and costs^132^. When merchants endorse or criticize merchants, recommend or disparage products, or hire review control agencies to generate multimodal fake reviews, they not only incur operational costs^133^ but also bear psychological and social costs. Users’ perceived psychological benefits also play a role in shaping their information-related behaviors. When merchants get psychological benefits, they are more likely to generate multimodal fake reviews by hiring a review control agency. Based on these reports, we hypothesize that: *perceived social costs negatively moderate relationship between supporting/disparaging merchants and multimodal fake review generation* (Hypothesis 9a); *perceived social costs negatively moderate relationship between recommending/disparaging products and multimodal fake review generation* (Hypothesis 9b); *perceived psychological benefits positively moderate relationship between supporting/disparaging merchants and multimodal fake review generation* (Hypothesis 10a); *perceived psychological benefits positively moderate relationship between recommending/disparaging products and multimodal fake review generation* (Hypothesis 10b).

### The moderating role of multimodal recognition and governance capabilities

Online multimodal fake review pertains to a diverse array of reviews, aligning with general notion of multimodal data. Purpose of multimodal identification is to discern instances of fake reviews. Multimodal review, in its various forms, holds greater utility value compared to unmoral online review^134^. Platform governance entails precise identification of multimodal reviews, followed by addressing hidden instances of multimodal fake reviews, ultimately ensuring an online ecosystem. Based on these reports, we hypothesize that: *multimodal recognition and governance capabilities negatively moderate relationship between supporting/disparaging merchants and multimodal fake review generation* (Hypothesis 11a); *multimodal recognition and governance capabilities negatively moderate relationship between recommending/disparaging products and multimodal fake review generation* (Hypothesis 11b); *multimodal recognition and governance capabilities negatively moderate relationship between perceptual behavior control and multimodal fake review generation* (Hypothesis 11c).

### User perception impact, merchant perception impact, and multimodal fake review generation

Analysis of shocks perceived in online multimodal reviews includes two perspectives: intensity and emotion. A higher perceived intensity of online multimodal review views corresponds to a greater number of existing reviews. An increase in number of existing online reviews encourages users to make purchases^135^. Furthermore, sentiment expressed in online book reviews has a significant influence on book sales^136^. Therefore, perceived impact of existing online multimodal reviews can influence fake review generation. Based on these reports, we hypothesize that: *user perception impact significantly and positively affects multimodal fake review generation* (Hypothesis 12a); *merchant perception impact significantly and positively affects multimodal fake review generation* (Hypothesis 12b).

**Figure 2:**
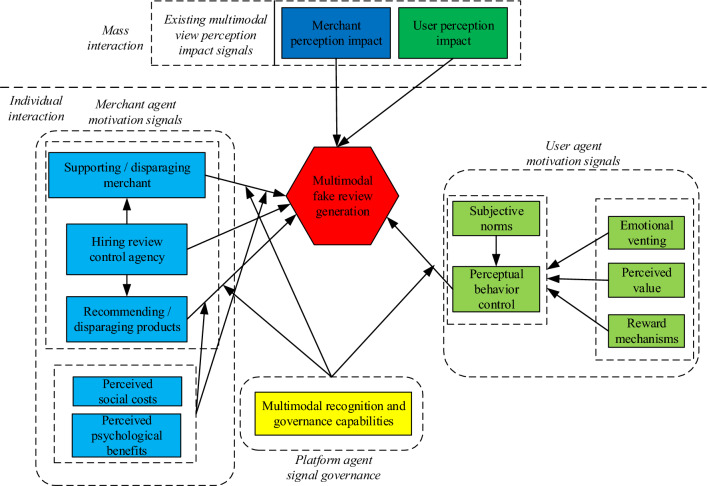
Research model. *Note*: red represents multimodal fake review generation, blue represents merchant-level feature factors, green represents user-level feature factors, and yellow represents platform-level feature factors.

Therefore, based on conceptual framework of multi-agent fake signal generation and hypotheses, Fig. 2 presents our research model.

Furthermore, upon review of relevant literature, we find that some scholars employ machine learning technique based on signaling theory to investigate impacts of electronic word of mouth as product signal on users’ purchase decisions^137,138^. Other researchers combine motivation theory with structural equation modeling to explore underlying motivations behind users’ additional review behavior in online reviews^139–141^. In addition, some scholars employ actor-network theory and complex networks analysis to examine influence of merchants and other agents on online review generation within online review environment^142,143^. Moreover, other scholars utilize information interaction theory and structural equation modeling to explore impacts of online reviews on user loyalty^144–146^. However, exploration of intrinsic mechanisms driving online fake review generation remains unclear, particularly in terms of integrating the theories of signaling, actor-network, motivation, and human–environment interaction hypothesis. Furthermore, there are few studies applying structural equation modeling, machine learning technique, and Bayesian complex networks analysis to explore intrinsic mechanisms of online fake review generation. Consequently, this study aims to address these gaps by integrating the theories of signaling, actor-network, motivation, and human–environment interaction hypothesis. Through incorporation of three methodologies, namely structural equation modeling, machine learning technique, and Bayesian complex networks analysis, this research seeks to find intrinsic mechanisms of online multimodal fake review generation, identify key determinants, and explore inherent correlations between these determinants as well as heterogeneity of male and female samples. This study can contribute to existing literature at both theoretical and methodological levels.

## Methods

### Study design

As three leading E-commerce platforms in China, Taobao, Jingdong, and Pinduoduo contain a considerable volume of fake reviews, it is essential to quantitatively analyze causal mechanisms behind fake review generation on these platforms. This analysis holds significant theoretical and practical implications for effectively preventing and managing fake reviews across all e-commerce platforms.

Based on the theories of signaling, actor-network, motivation, and human–environment interaction hypothesis, we develop a theoretical model for online multimodal fake review generation mechanisms. Our model encompasses platforms, users, and merchants. To gather data for our model, we design a questionnaire that consists of two parts. The first part collects basic information from participants, consisting of 9 items. The second part measures variables in model, with a total of 42 original items designed for explanatory and interpreted variables. We distribute electronic questionnaires to users of China’s three leading E-commerce platforms (Taobao, Jingdong, and Pinduoduo). We choose users from these platforms as survey respondents because they have extensive online shopping experience and frequently engage in online shopping reviews. Thus, samples from these platforms are highly representative of studying issue of fake reviews and can provide credible experimental results. Specifically, user samples from these three platforms offer strong data support for drawing trustworthy experimental conclusions. We obtain 1500 valid samples in total (500 valid samples from Taobao, Jingdong, and Pinduoduo platforms, respectively).

The following section compares the approaches used most in previous related research with the techniques and ideas of this research. Table 1 presents comparative analysis of research methodologies employed in relevant research.

**Table 1:** Comparative analysis of methodologies employed in relevant research.

Title	Author	Date	Topic	Methods
Impacts of consumer cognitive process to ascertain online fake review: a cognitive dissonance theory approach	Chatterjee et al.^147^	2023	Fake review	Structural equation modeling
Leveraging SMEs technologies adoption in the Covid‑19 pandemic: a case study on Twitter‑based user‑generated content	Saura et al.^148^	2023	User generated content	Structural equation modeling
Exploring the influence of emotionality and expertise on online wine reviews: does greater knowledge lead to less review?	Qi et al.^149^	2024	Online review	Structural equation modeling
The role of positive online reviews in risk-based consumer behaviors: an information processing perspective	Lam et al.^150^	2023	Online review	Structural equation modeling
Do live streaming and online consumer reviews jointly affect purchase intention?	Qin et al.^151^	2023	Online review	Structural equation modeling
A comprehensive survey on machine learning approaches for fake news detection	Alghamdi et al.^152^	2023	Fake review	Machine learning
Design of an efficient integrated feature engineering based deep learning model using CNN for customer’s review helpfulness prediction	Sharma et al.^153^	2024	E-commerce	Deep learning
Assessing the helpfulness of hotel reviews for information overload: a multi‑view spatial feature approach	Liu et al.^154^	2024	Online review	Deep learning
Machine learning-based e-commerce platform repurchase customer prediction model	Liu et al.^155^	2020	E-commerce	Machine learning
Helpfulness of online reviews: examining review informativeness and classification thresholds by search products and experience products	Sun et al.^156^	2019	Online review	Machine learning
Ranking online consumer reviews	Saumya et al.^157^	2018	Online review	Machine learning
Analysis of customers’ satisfaction with baby products: the moderating role of brand image	Nilashi et al.^158^	2023	E-commerce	Machine learning, structural equation modeling
Enhancing the prediction of user satisfaction with metaverse service through machine learning	Hong Lee et al.^159^	2022	E-commerce	Machine learning, structural equation modeling
Revealing travellers’ satisfaction during COVID-19 outbreak: moderating role of service quality	Nilashi et al.^160^	2022	Online review	Machine learning, structural equation modeling
The role of consumer reviews in e-commerce platform credit supervision: a signaling game model based on complex network	Xu et al.^161^	2024	Online review	Complex network analysis
Sentiment mining of online reviews of peer-to-peer accommodations: customer emotional heterogeneity and its influencing factors	Li et al.^162^	2023	Online review	Social network analysis
Game theory based emotional evolution analysis for chinese online reviews	Bu et al.^163^	2016	Online review	Social network analysis
Learning user sentiment orientation in social networks for sentiment analysis	Chen et al.^164^	2022	E-commerce	Complex network analysis
Investigating transitive influences on WOM: from the product network perspective	Chen et al.^165^	2016	E-commerce	Complex network analysis
Integrating node centralities, similarity measures, and machine learning classifiers for link prediction	Anand et al.^166^	2022	E-commerce	Machine learning, complex network analysis

According to Table 1, most of similar literature employs methodologies such as structural equation modeling, machine learning technique, and complex network analysis to investigate related issues. Consequently, this study aims to address limitations of existing research by innovatively integrating three methods: first, employing structural equation modeling to explore intrinsic causal mechanisms behind online multimodal fake review generation, thereby uncovering black box of causal process of online multimodal fake review generation; second, employing machine learning to quantitatively analyze importance of determinants that influence online multimodal fake review generation, thus identifying key determinants of online multimodal fake review generation; finally, employing Bayesian complex networks analysis to delve into inherent correlations between these determinants and heterogeneity of male and female samples.

In summary, using survey data from users of China’s three leading E-commerce platforms (Taobao, Jingdong, and Pinduoduo), first, we employ structural equation modeling to examine mechanisms of online multimodal fake review generation, including measurement model analysis, structural model analysis, moderating effects analysis, and mediating effects analysis. Second, building on result of structural equation modeling, we use machine learning technique to further analyze key feature factors of online multimodal fake review generation and divide them into distinct regimes. Finally, based on results of machine learning technique, we investigate intrinsic correlations of these feature factors through Bayesian complex networks analysis, including network centrality analysis of full sample, male sample and female sample.

**Figure 3:**
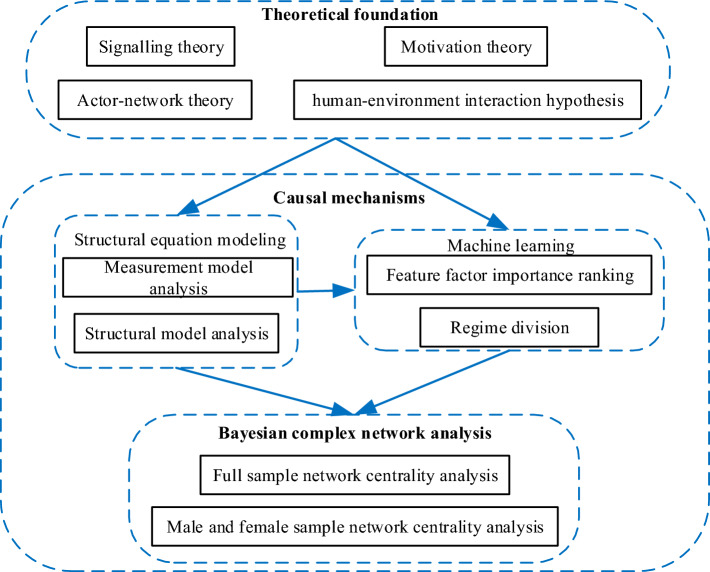
Proposed research flow of our work.

Therefore, Fig. 3 presents proposed research flow of our work.

### Participants

We obtain survey data from users of China’s three leading E-commerce platforms (Taobao, Jingdong, and Pinduoduo) from 15 March 2023 to 25 June 2023. Users of these three platforms have extensive online shopping experience and they frequently participate in online shopping reviews. Therefore, a sample of users from these platforms provides strong data support to draw credible experimental conclusions. Before distributing a large number of questionnaires, we conduct a pre-survey to correct terminology difficulties, improve question clarity, and enhance question differentiation. Moreover, we exclude questions that are irrelevant^167^. After eliminating any duplicate responses, we obtain a total of 1500 valid questionnaires. Sample contains 500 questionnaires from Taobao, 500 questionnaires from Jingdong, and 500 questionnaires from Pinduoduo, respectively. Table 2 presents descriptive statistics for demographic variables.

**Table 2:** Descriptive statistics for demographic variables.

Variables	Variable definitions	Effective percentage	Variables	Variable definitions	Effective percentage
City type	First-tier city	28.53	Monthly income after tax	≤ 2000	53.94
	New first-tier city	13.80		2000–7000	35.73
	Second-tier city	6.07		> 7000	10.33
	Third-tier city	39.07	Years of purchase experience in online	≤ 1	1.40
	Fourth-tier city	9.07		1–3	40.47
	Fifth-tier city	3.46		> 3	58.13
Gender	Male	53.67	Number of online reviews in the past year	0	32.60
	Female	46.33		1–3	12.53
Age	28 years and under	58.00		4–8	19.47
	29–40 years old	23.47		> 8	35.40
	41years and above	18.53	Region	Eastern China	23.27
Academic qualifications	High school and below	0.33		Southern China	37.13
	Associate degree	49.74		Western China	7.80
	Bachelor’s degree	39.47		Northern China	20.93
	Master’s degree	10.13		Central China	10.73
	Doctoral Degree	0.33		Hong Kong, Macau, and Taiwan	0.07
Occupation	Worker	29.00		Overseas regions	0.07
	Student	53.33			
	Unemployed	17.67			
	Retired	0.00			

### Ethical approval

All procedures performed in studies involving human participants are in accordance with the ethical standards of the institutional and/or national research committee and with the 1964 Helsinki declaration and its later amendments or comparable ethical standards.

### Informed consent

Informed consent is obtained from all subjects involved in the study. All materials and methods are performed in accordance with the instructions and regulations and this research has been approved by a committee at Nanchang University, China.

### Measures

To ensure scientificity and robustness of our questionnaire, final questionnaire consists of two sections. Section (i) is about personal and professional characteristics of respondents, and section (ii) is about measuring variables of theoretical framework to measure emotional venting (3 items), perceived value (3 items), reward mechanisms (2 items). subjective norms (3 items), perceptual behavior control (3 items), supporting/disparaging merchants (3 items), recommending/disparaging products (3 items), hiring review control agency (2 items), perceived social costs (2 items), perceived psychological benefits (2 items), multimodal recognition and governance capabilities (2 items), user perception impact (2 items), merchant perception impact (2 items), and multimodal fake review generation (3 items). A seven-point Likert scale (1-completely disagree to 7-completely agree) to measure items from survey respondents’ perspectives. Table 3 presents final survey items and relevant references.

**Table 3:** Final survey items and relevant references.

Construct	Statement	References
Emotional venting	I will overly brag in reviews to express inner pleasure	Kim et al.^168^
	I will be overly denigrating in reviews to express inner displeasure	
	I may give multiple aspects of item positive reviews to express inner satisfaction	
Perceived value	When I think an item has value, I will give overly positive reviews	Sheth et al.^169^
	When I think an item has no value, I will give overly negative reviews	
	When I think an item has value, I may give multiple aspects of item positive reviews	
Reward mechanisms	When merchants guide users to make positive reviews through positive feedback, some reviews contain characteristic signs such as “positive feedback”	Henning-Thurau et al.^170^
	To get rewarded by merchants, my reviews are positive	
Subjective norms	If a relative, friend or classmate suggests reviews of an item, I will give reviews	Dixit et al.^171^
	If platform suggests reviews of an item, I will give reviews	
	If a merchant suggests reviews of an item, I will give reviews	
Perceptual behavior control	I have enough patience to review products	Elliott et al.^172^
	I can decide whether or not to review products	
	I have some experience in reviewing products	
Supporting/disparaging merchants	When I am satisfied with a product, I will write overly positive reviews to support merchant	Henning-Thurau et al.^170^
	When I am dissatisfied with a product, I will write excessively negative reviews to discredit merchant	
	When I am dissatisfied with a product, I will give biased bad reviews due to non-quality determinants of product	
Recommending/disparaging products	Most of reviews show richer emotions and are somewhat provocative	Kim et al.^168^
	Most reviews are overly positive and contain large extremely complimentary words	
	Most of reviews are expressions of evaluator's personal feelings, and expressions are too denigrating	
Hiring review control agency	I feel that merchants can hire agents to generate multimodal fake reviews, reviews are general descriptions of products	Lawrence et al.^173^
	A multimodal fake review generated by agency covers workmanship, sizing, packaging, and logistics of product	
Perceived social costs	I think merchants have enough energy to generate multimodal fake reviews	Dreber et al.^174^
	I think costs have an impact on whether or not merchants generate multimodal fake reviews	
Perceived psychological benefits	I think merchants believe that generating multimodal fake reviews can bring revenue	Leonidou et al.^175^
	I think merchants will consider cost–benefit of generating multimodal fake reviews	
Multimodal recognition and governance capabilities	I believe platforms are capable of recognizing multimodal fake reviews	Chaturvedi et al.^176^
	I believe platforms are capable of governing multimodal fake reviews	
User perception impact	The greater/smaller number of multimodal reviews, the greater/smaller probability that I will rate product positively	Park et al.^177^
	A product has multimodal reviews the more positive/negative emotions, the greater/smaller probability that I will give a positive review	
Merchant perception impact	I believe the greater/smaller number of existing multimodal reviews, the lower/greater probability that merchant generates multimodal fake reviews	Van Slyke et al.^178^
	I think the more positive/negative emotions there are already multimodal reviews, the lower/greater probability that merchant generates multimodal fake reviews	
Multimodal fake review generation	I used to generate modal fake reviews for some of above motives	Lawrence et al.^173^
	My family, friends, and classmates used to generate modal fake reviews for one of above motives	
	Most multimodal fake reviews are generated for some of above motives	

### Data analysis

Structural equation modeling (SEM) technique is used to test our research model. SEM is a multivariate statistical analysis method used to analyze relationships between constructs with multiple items. Two basic statistical methods are used for testing SEM: covariance-based SEM and variance-based partial least square (PLS). Covariance-based modeling is suitable for model validation and comparison, while PLS is used for complex structural models with a large number of constructs^179^, hence use of covariance-based SEM in our study. Two-step approach of Liu et al.^180^ is used to carry out SEM. The first step is to perform a confirmatory factor analysis (CFA) to obtain a satisfactory measurement model, and the second is to develop a structural model and test it. Additionally, based on results of SEM, according to the study of Wang et al.^181^, we employ GBM (Gradient Boosting Regression) package in R to analyze importance of feature factors that influence online multimodal fake review generation. Furthermore, according to the study of Williams et al.^182^, we utilize BGGM (Bayesian Gaussian Graphical Models) package in R to examine inherent correlations among feature factors in full sample and analyze heterogeneity of male and female samples.

## Results

### Structural equation modeling analysis

In this section, this paper employs structural equation techniques to identify factors that influence online multimodal fake review generation and analyze their impact pathways. Analysis includes reliability examinations, model fit indexes calculating, and hypotheses testing.

First, reliability examinations. In this paper, we refer to scholars Saadati et al.^183^ and conduct validation determinants analysis to analyze reliability and validity of measurement model. As a result of analysis, observed variables with standardized determinants loadings below 0.670 are excluded from our study. Validation determinants analysis shows that standard error (S.E.) of each observed variable under unstandardized estimation of our model is greater than 0, indicating absence of any covariance problem among observed variables. Determinants loadings of each observational variable are found to be significant (*p* < 0.001, |t|> 1.96). Standardized loading estimates of each observational indicator range from 0.668 to 0.942, all above 0.6. Conformal combination reliability CR ranges from 0.769 to 0.876, all above 0.7. Average coefficient of variation extract AVEs is also above 0.5, suggesting good convergent validity for model constructed in this paper. Moreover, square root of AVE for each observed variable exceeds correlation coefficient between variables, indicating good discriminant validity for our model. Thus, our model proposed in this paper is an acceptable model.

Second, model fit indexes calculating. We compute fit indexes of structural and measurement models, and Table 4 presents calculation results.

**Table 4:** Fit indexes.

Indexes	Measurement model	Structural model	Suggested values
Chi-square/df	2.736	2.869	< 3
Comparative fit index	0.984	0.963	> 0.9
Goodness of fit index	0.952	0.906	> 0.9
Adjusted goodness-of-fit index	0.916	0.909	> 0.8
Root mean square error of approximation	0.013	0.052	< 0.08
Tucker Lewis index	0.942	0.928	> 0.9
Normed fit index	0.939	0.927	> 0.9

According to Table 4, values of model fit indexes, including chi-square /df, comparative fit index, goodness of fit Index, adjusted goodness-of-fit index, root mean square error of approximation, tucker lewis index, and normed fit index, are greater than suggested values for both measurement and structural models. Therefore, based on study of Nan et al.^184,185^, Kar et al.^186^, and Shahzad et al.^187^, values of indicators are considered acceptable.

Finally, hypotheses testing. We test model paths using a significance level of *p* = 0.05. Upon examining path coefficient estimates, hypothesized path 4c is not significant and we remove it. After modificating our model, we retest constructed model’s impact paths, Table 5 presents final test results.

**Table 5:** Final paths test results.

Assumptions	Standardized coefficient	S.E	C.R	*P*
Hypothesis 1a: emotional venting → perceptual behavior control	0.135	0.019	5.004	***
Hypothesis 1b: perceived value → perceptual behavior control	0.097	0.023	3.710	***
Hypothesis 1c: reward mechanisms → perceptual behavior control	0.204	0.031	8.023	***
Hypothesis 1d: subjective norms → perceptual behavior control	0.141	0.026	5.376	***
Hypothesis 4a: hiring review control agency → supporting/disparaging merchants	0.113	0.043	4.410	***
Hypothesis 4b: hiring review control agency → recommending/disparaging products	0.200	0.034	7.912	***
Hypothesis 2: perceptual behavior control → multimodal fake review generation	0.058	0.028	2.570	**
Hypothesis 5: supporting/disparaging merchants → multimodal fake review generation	0.317	0.020	14.154	***
Hypothesis 7: recommending/disparaging products → multimodal fake review generation	0.123	0.025	5.498	***
Hypothesis 12a: user perception impact → multimodal fake review generation	0.132	0.026	4.382	***
Hypothesis 12b: merchant perception impact → multimodal fake review generation	0.242	0.031	8.008	***

**p < 0.01, ***p < 0.001.

According to Table 5, hypotheses 1a–1d, hypothesis 2, hypothesis 4a, hypothesis 4b, hypothesis 5, hypothesis 7, hypothesis 12a, and hypothesis 12b are supported. First, emotional venting, perceived value, reward mechanisms, and subjective norms exhibit significant positive indirect effects on multimodal fake review generation. Additionally, perceptual behavior control demonstrates direct and significant positive effects on multimodal fake review generation. Second, hiring review control agency directly and significantly influences both supporting/disparaging merchants, as well as recommending/disparaging products. Moreover, both supporting/disparaging merchants and recommending/disparaging products directly and significantly contribute to multimodal fake review generation. Finally, both user perception impact and merchant perception impact directly and significantly influence multimodal fake review generation.

Furthermore, according to the study of Huifeng et al.^188^, we adopt hierarchical moderated regression analysis to evaluate moderating effect of platforms’ multimodal recognition and governance capabilities. Table 6 presents test results.

**Table 6:** Test results of moderating effects.

Interaction term	β	VIF
Supporting/disparaging merchants × Multimodal recognition and governance capabilities	− 0.109*** (4.723)	1.016
Recommending/disparaging products × Multimodal recognition and governance capabilities	− 0.011 (− 0.425)	1.008
Perceptual behavior control × Multimodal recognition and governance capabilities	− 0.007 (0.290)	1.019
Supporting/disparaging merchants × Perceived social costs	− 0.005 (− 0.238)	1.003
Recommending/disparaging products × Perceived social costs	− 0.093*** (3.873)	1.041
Supporting/disparaging merchants × Perceived psychological benefits	0.001 (− 0.065)	1.027
Recommending/disparaging products × Perceived psychological benefits	0.041 (1.758)	1.006

***p < 0.001, with corresponding t-values in parentheses.

**Figure 4:**
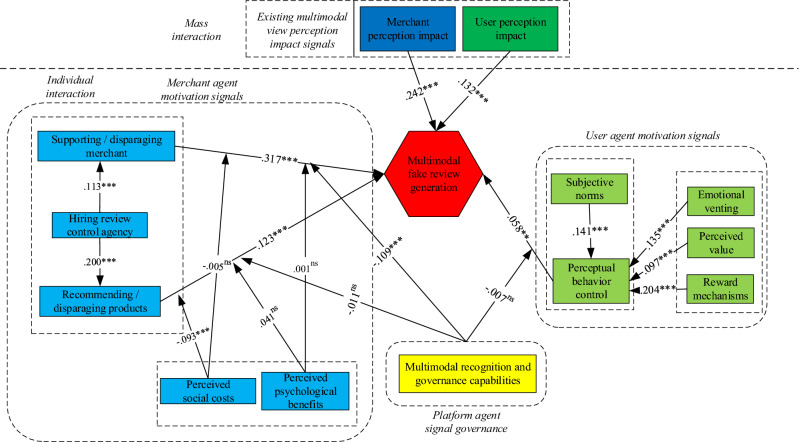
Final results of model paths and moderating effects testing. *Note*: *P < 0.05, **P < 0.01, ***P < 0.001, *ns* indicates not significant, Red represents multimodal fake review generation, blue represents merchant-level feature factors, green represents user-level feature factors, and yellow represents platform-level feature factors.

According to Table 6, hypotheses 9b and 11a are supported. First, perceived social costs exert a significant weakening influence on positive correlation between recommending/disparaging products and multimodal fake review generation. Second, platforms’ multimodal recognition and governance capabilities significantly inhibit positive relationship between supporting/disparaging merchants and multimodal fake review generation. Finally, perceived psychological benefits exert insignificant moderating effects on merchants’ multimodal fake review generation. Figure 4 presents final results of model paths and moderating effects tests.

In addition, this study employs Bootstrap method to examine mediating role of perceptual behavior control, supporting/disparaging merchants and recommending/disparaging products. More specifically, according to the study of Kim et al.^189^, mediating effects are assessed based on Bootstrap method, so this paper selects a sample size of 5000 to test mediation effects at a 95% confidence interval. Table 7 presents details analysis results.

**Table 7:** Test results of mediating effects. (Bootstrap = 5000).

Mediation effects			
Model pathways	Boot SE	Boot LLCI	Boot ULCI
Hiring review control agency → (Supporting/disparaging merchants) → Multimodal fake review generation	0.0188	0.0440	0.1191
Hiring review control agency → (Recommending/disparaging products) → Multimodal fake review generation	0.0132	0.0446	0.0972
Emotional venting → (Perceptual behavior control) → Multimodal fake review generation	0.0064	0.0046	0.0298
Perceived value → (Perceptual behavior control) → Multimodal fake review generation	0.0065	0.0090	0.0341
Reward mechanisms → (Perceptual behavior control) → Multimodal fake review generation	0.0153	0.0456	0.1056
Subjective norms → (Perceptual behavior control) → Multimodal fake review generation	0.0099	0.0172	0.0566

Direct effects			
Model pathways	SE	LLCI	ULCI
Hiring review control agency → (Supporting/disparaging merchants) → Multimodal fake review generation	0.0366	− 0.0749	0.0687
Hiring review control agency → (Recommending/disparaging products) → Multimodal fake review generation	0.0407	− 0.0701	0.0895
Emotional venting → (Perceptual behavior control) → Multimodal fake review generation	0.0221	0.2824	0.3691
Perceived value → (Perceptual behavior control) → Multimodal fake review generation	0.0273	0.3773	0.4844
Reward mechanisms → (Perceptual behavior control) → Multimodal fake review generation	0.0425	− 0.0340	0.1327
Subjective norms → (Perceptual behavior control) → Multimodal fake review generation	0.0333	0.2462	0.3768

According to Table 7, both mediating effects of hiring review control agency on multimodal fake review generation do not include 0 ([0.0440, 0.1191], [0.0446, 0.0972]). Therefore, supporting/disparaging merchants and recommending/disparaging products serve as mediating variables between hiring review control agency and multimodal fake review generation. However, two direct effects of hiring review control agency on multimodal fake review generation do not exist (intervals of [− 0.0749, 0.0687] and [− 0.0701, 0.0895] include 0). Consequently, supporting/disparaging merchants and recommending/disparaging products fully mediate relationship between intermediary of hiring review control agency and multimodal fake review generation. Moreover, mediating effect between emotional venting and multimodal fake review generation does not include 0 ([0.0046, 0.0298]). Therefore, perceptual behavior control serves as a mediating variable between emotional venting and multimodal fake review generation. However, a direct effect of emotional venting on multimodal fake review generation exists (interval [0.2824, 0.3691] does not include 0). As a result, perceptual behavior control partially mediates relationship between emotional venting and multimodal fake review generation. Similarly, perceptual behavior control partially mediates relationship between perceived value and multimodal fake review generation, as well as between subjective norms and multimodal fake review generation. Additionally, mediating effect between reward mechanisms and multimodal fake review generation does not include 0 ([0.0456, 0.1056]). Hence, perceptual behavior control serves as a mediating variable between reward mechanisms and multimodal fake review generation. However, a direct effect of reward mechanisms on multimodal fake review generation does not exist (interval [− 0.0340, 0.1327] includes 0). Consequently, perceptual behavior control fully mediates relationship between reward mechanisms and multimodal fake review generation.

### Machine learning prediction

In previous section, we use structural equation modeling to analyze online multimodal fake review generation mechanism, but we are unable to quantify weights of factors affecting online multimodal fake review generation, and we do not discover key feature factors affecting online multimodal fake review generation. Therefore, according to the study of Wang et al.^181^, this study further quantify weights of factors affecting online multimodal fake review generation and divide them into distinct regimes using GBM (Gradient Boosting Regression) package in R, to discover key feature factors affecting online multimodal fake review generation, and to further open up black box of online multimodal fake review generation mechanism.

First, sample division. According to the study of Moussa et al.^190^, Training set includes 960 sample data, validation set includes 240 sample data, and test set includes 300 sample data.

**Figure 5:**
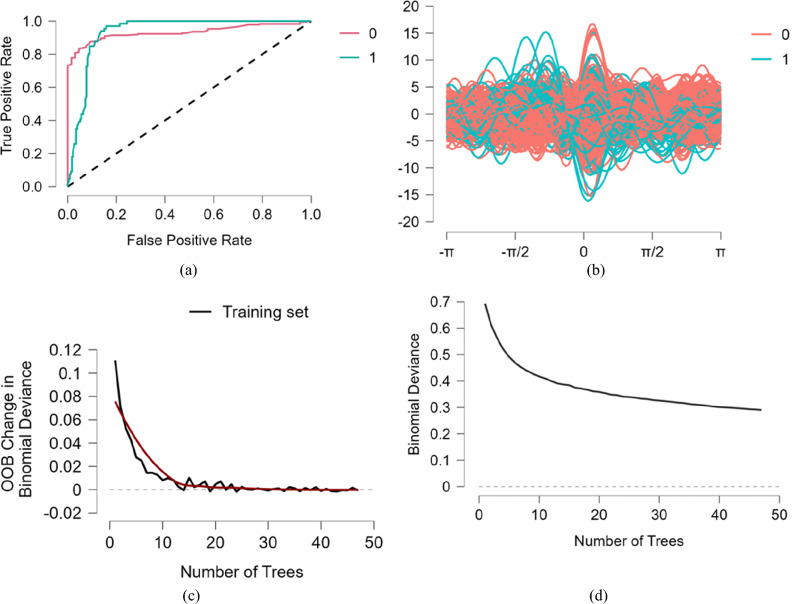
ROC curve, Andrews curves, model training set accuracy, and prediction error. *Note*: (*a*) is ROC curves, (*b*) is Andrews curves, (*c*) is model training set accuracy, and (*d*) is model prediction error.

Second, model prediction performance evaluation. According to the study of Spee et al.^191^, Accuracy of model is evaluated using Eq. (1), Eq. (1) calculates ratio of correct predictions to all predictions. Correct predictions include true positive (TP) and true negative (TN) classes, while predictions themselves consist of true positives (TP), false positives (FP), true negatives (TN), and false negatives (FN). As mentioned in Eq. (2), precision measures proportion of true positive predictions (TP) relative to all positive predictions, including both true positives (TP) and false positives (FP). On the other hand, recall quantifies percentage of true positive predictions (TP) out of all positive instances in testing dataset, considering both true positives (TP) and false negatives (FN) as shown in Eq. (3). F1-value, as demonstrated in Eq. (4), is harmonic mean of precision and recall. It is calculated by multiplying precision and recall by two and then dividing result by their sum. Table 8 presents model evaluation metrics, Fig. 5a,b present Receiver Operating Characteristic (ROC) curves and Andrews curves. True Positive Rate (TPR) on vertical coordinate of ROC curve represents proportion of actual positive instances among all positive instances in predicted positive class. Similarly, False Positive Rate (FPR) on horizontal coordinate represents proportion of actual negative instances among all negative instances in predicted positive class. In this context, “1” denotes a positive class, while “0” denotes a negative class. True Positive Rate (TPR) is evaluated using Eq. (5), and False Positive Rate (FPR) is evaluated using Eq. (6). Additionally, AUC value represents area under ROC curve. A higher AUC value indicates better predictive performance of model in terms of classification. Figure 5c,d present training set accuracy, and changes in prediction error as regression tree increases. “0” denotes absence of multimodal fake review generation, and “1” denotes presence of multimodal fake review generation.

1 $$ Accuracy{ = }\frac{TP + TN}{{TP + FP + TN + FN}} $$

2 $$ Precision{ = }\frac{TP}{{TP + FP}} $$

3 $$ Recall{ = }\frac{TP}{{TP + FN}} $$

4 $$ F1 - value{ = 2}\frac{precision \times recall}{{precision{ + }recall}} $$

5 $$ TPR{ = }\frac{TP}{{TP + FN}} $$

6 $$ FPR = \frac{FP}{{FP + TN}} $$

**Table 8:** Model evaluation metrics.

Evaluation indicators	Average/total	Evaluation indicators	Average/total
Accuracy	0.884	F1—value	0.884
Recall rate	0.883	AUC	0.932

According to Table 8, recall, F1 score, and AUC achieve values greater than 0.85. According to Fig. 5a,b, “0” and “1” curves reside within realm of meaningful sensitivity, they deviate from reference line, and model’s classification performance is also apparent. According to Fig. 5c,d, as number of regression trees increases, model’s loss function continues to decrease, indicating that training accuracy is improving, and prediction error is gradually reducing. Therefore, all evaluation metrics demonstrate a good predictive performance of our model.

**Figure 6:**
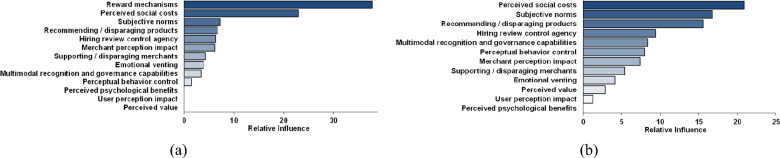
Feature factors ranking & Robustness test result.

Third, feature factor importance ranking and regime division. Table 9 and Fig. 6a present results of relative importance ranking analysis for feature factors. Table 10 presents results of regime division. Moreover, we get four conclusions: (1) reward mechanisms are key feature factor of online multimodal fake review generation. This implies that material incentives serve as driving force behind users’ generation of such deceptive content. Merchants’ reward information holds a significant influence over online multimodal fake review generation, particularly in context of user interactions with complex information. From a policy perspective, this discovery holds substantial practical value. Future governmental policies should focus on regulating incentivization strategies like cashback offers for positive reviews. Such measures can deter unscrupulous merchants from inducing users to generate multimodal fake reviews, ultimately fostering a healthier internet ecosystem; (2) perceived social costs significantly influence online multimodal fake review generation, whereas perceived psychological benefits exhibit no such impact. This finding further validates outcomes of previous structural equation modeling analysis: perceived psychological benefits do not exert a significant moderating effect on merchants’ multimodal fake review generation. This suggests that merchants are influenced by social costs, and display risk-averse behavior^192^, thereby exhibiting risk-averse tendencies; (3) user-level motivational determinants exert a greater impact compared to merchant-level motivational determinants. Importance ratio of all feature factors about user agents is found to be 50.317%, whereas that of merchant agents is 40.13%. This discrepancy may be attributed to fact that users generate fake reviews at almost no cost, Conversely, merchants that generate fake reviews have to bear social cost of pressure; (4) we can divide feature factors into four regimes. The first regime includes reward mechanisms and perceived social costs. The second regime includes subjective norms, recommending/disparaging products, hiring review control agency, and merchant perception impact. The third regime includes supporting/disparaging merchants, emotional venting, multimodal recognition and governance capabilities, and perceptual behavior control. The fourth regime includes perceived psychological benefits, user perception impact, and perceived value.

**Table 9:** Feature factors ranking.

Type of information interaction	Ranking	Relative importance
Individual interaction		
Reward mechanisms (user)	1	37.783
Perceived social costs (merchant)	2	22.917
Subjective norms (user)	3	7.227
Recommending/disparaging products (merchant)	4	6.650
Hiring review control agency (merchant)	5	6.294
Supporting/disparaging merchants	7	4.269
Emotional venting (user)	8	3.837
Multimodal recognition and governance capabilities (platform)	9	3.401
Perceptual behavior control (user)	10	1.470
Perceived psychological benefits (merchant)	11	0.000
Perceived value (user)	13	0.000
Mass interaction		
Merchant perception impact	6	6.151
User perception impact	12	0.000

**Table 10:** Regime division.

Regime	Feature factor	Relative importance	Regime	Feature factor	Relative importance
Regime 1	Reward mechanisms	37.783	Regime 3	Supporting/disparaging merchants	4.269
	Perceived social costs	22.917		Emotional venting	3.837
Regime 2	Subjective norms	7.227		Multimodal recognition and governance capabilities	3.401
	Recommending/disparaging products	6.650		Perceptual behavior control	1.470
	Hiring review control agency	6.294	Regime 4	Perceived psychological benefits	0.000
	Merchant perception impact	6.151		User perception impact	0.000
				Perceived value	0.000

Finally, robustness test. To ensure robustness of our empirical findings, according to the study of Jain et al.^193^, we remove the most significant feature factor, namely “reward mechanisms”, to examine remaining feature factors’ importance ranking. Figure 6b presents test result.

Upon comparing Fig. 6a,b, in general, robustness test result aligns with previous benchmark findings. This is because top five feature factors remain unchanged, and following elimination of pivotal “reward mechanisms”, perceived social costs are still the second most important determinant of online multimodal fake review generation. Moreover, final three feature factors remain unchanged. Hence, feature factors’ importance ranking is robust.

### Bayesian complex networks analysis

In previous section, we employ structural equation modeling to establish a causal relationship between feature factors influencing online multimodal fake review generation. Additionally, we utilize machine learning technique to explore importance of each feature factor. However, we are unable to find inherent associations between these feature factors. Therefore, to develop a comprehensive understanding of inherent relationships between variables that influence online multimodal fake review generation, according to the study of Williams et al.^182^, we investigate significance of these variables and their associations, as well as heterogeneity of male and female samples through BGGM (Bayesian Gaussian Graphical Models) package in R, to conduct Bayesian complex networks analysis, including network centrality analysis of full sample, male sample, and female sample. From perspective of probability theory^194^, a Bayesian complex network represents joint distribution of a set of random variables, according to chain rule and conditional independence, joint distribution of a series of random variables $X = \{ X_{1} , \ldots ,X_{n} \}$ can be written as Eq. (7). Variables’ connections are defined as network links. We employ R language with BGGM (Bayesian Gaussian Graphical Models) package to conduct complex networks analysis. This package allows for fitting of Bayesian Gaussian Graphical Models, facilitating hypothesis testing, estimation, and validation. Additionally, this package enables comparisons between Gaussian graphical models and prediction of individual nodes^195^.

7 $$ P(X_{1} , \ldots ,X_{{\text{n}}} ) = P(X_{1} )P(X_{2} |X_{1} ) \ldots P(X_{{\text{n}}} |X_{1} ,X_{2} , \ldots ,X_{{{\text{n}} - 1}} ) = \prod\nolimits_{1}^{{\text{n}}} {P(X_{i} |\pi (X_{i} ))} $$

Note: $\pi (X_{i} )$ is collection of parent of $X_{i} ,\pi (X_{i} ) \subseteq \{ X_{1} , \ldots ,X_{i - 1} \}$, given value of $\pi (X_{i} )$; $X_{i}$ is conditionally independent of other variables^196^ in $\{ X_{1} , \ldots ,X_{i - 1} \}$.

**Figure 7:**
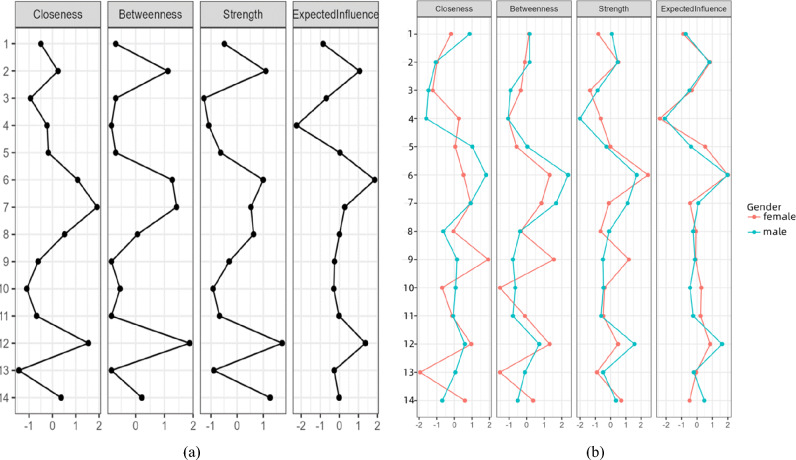
Network centrality analysis of full sample, male sample and female sample of online multimodal fake review generation. *Note*: sample size N = 1500; in (**a**), 1—multimodal fake review generation; 2—merchant perception impact; 3—user perception impact; 4—multimodal recognition and governance capabilities; 5—perceived psychological benefits; 6—perceived social costs; 7—hiring review control agency; 8—supporting/disparaging merchants; 9—recommending/disparaging products; 10—perceptual behavior control; 11—subjective norms; 12—reward mechanisms; 13—perceived value; 14—emotional venting; in (**b**), 1—multimodal fake review generation; 2—merchant perception impact; 3—user perception impact; 4—multimodal recognition and governance capabilities; 5—perceived psychological benefits; 6—perceived social costs; 7—hiring review control agency; 8—supporting/disparaging merchants; 9—recommending/disparaging products; 10—perceptual behavior control; 11—subjective norms; 12—reward mechanisms; 13—perceived value; 14—emotional venting.

Network centrality analysis of full sample, male sample and female sample. Network centrality value primarily signifies position and role of each node in network. Our focus revolves around four key aspects: closeness centrality, betweenness centrality, strength centrality, and expected influence centrality^197^. Figure 7a,b present network centrality analysis of full sample, male sample and female sample of online multimodal fake review generation.

Closeness centrality is a measure that quantifies average shortest path length between a node and all other nodes in a given network^198^. This measure indicates level of influence that a node has in generating multimodal fake reviews. A higher value signifies a greater degree of centrality and a closer proximity to other nodes. Based on the study of Elmezain et al.^199^, closeness centrality value is evaluated using Eq. (8). According to Fig. 7a, in full sample, hiring review control agency, reward mechanisms, and perceived social costs exhibit higher values of centrality. This suggests that they have stronger connections with other nodes and occupy a more intermediate position in network. Consequently, fake review prevention efforts should prioritize these determinants. According to Fig. 7b, in both male and female sample, closeness centrality values of reward mechanisms, perceived social costs, and hiring review control agency are all higher, aligning with findings of analysis conducted on full sample. However, male sample exhibits a higher closeness centrality value for perceived social costs compared to female sample. This indicates that men tend to be more rational in considering costs involved when generating online multimodal fake reviews.

8 $$ Closeness\,centrality\,value{ = }\frac{{n - {1}}}{{\sum\limits_{v \in V} {d(u,v)} }} $$

Note: $d(u,v)$ is the shortest path length from node $u$ to node $v$.

Betweenness centrality measures effectiveness of a node in acting as a bridge on the shortest path between two other nodes^200^. Nodes with higher betweenness centrality values have greater control in network. Based on the study of Liu et al.^201^, betweenness centrality value is evaluated using Eq. (9). According to Fig. 7a,b, in both full sample, male sample, and female sample, all nodes in online multimodal fake review generation exhibit non-zero betweenness centrality. This indicates that every node in network acts as a “bridge” connecting and influencing other nodes.

9 $$ Betweenness\,centrality\,value{ = }\sum\limits_{s \ne v \ne t} {\frac{{\sigma_{st} (v)}}{{\sigma_{st} }}} $$

Note: $v$ is node that we want to compute betweenness centrality, $s$ and $t$ are two other distinct nodes in network, $\sigma_{st}$ is total number of shortest paths from node s to node t, and $\sigma_{st} (v)$ is number of paths passing through node v from node s to node t in the shortest path.

Strength centrality is a natural generalization of node degree in a powerless network. Sum of weights of edges that are directly associated with a given node determines strength centrality^202^. A higher value of strength centrality indicates a stronger direct connection of node with other nodes. Based on the study of Abbasi et al.^203^, strength centrality value is evaluated using Eq. (10). Figure 7a demonstrates that reward mechanisms exhibit the largest value of strength centrality, suggesting that they have the greatest node strength in network and the most direct connections with other nodes. User perception impact demonstrates the smallest value of strength centrality, indicating that it has the most indirect connections to other nodes. This observation further reinforces significance of reward mechanisms as a key node in network. Moreover, intervening in reward mechanisms can have a substantial impact on effectiveness of fake review governance. Additionally, according to Fig. 7b, strength centrality values of perceived social costs are higher in both male and female sample, indicating that perceived social costs play a significant role in online multimodal fake review generation.

10 $$ strength\,centrality\,value = \sum\limits_{u \in N(v)} {w_{uv} } $$

Note: $N(v)$ denotes set of nodes directly connected to node v, i.e., neighbors of node v, and $w_{uv}$ denotes weights of edges between node u and node v.

Expected influence centrality is used to describe magnitude of a node’s influence on network^204^. A larger value of expected influence signifies a greater influence on entire social relationship network. Based on the study of Schmidt et al.^205^, expected influence centrality value is evaluated using Eq. (11). According to Fig. 7a, in full sample, variables with the highest positive expected impact on online multimodal fake review generation are reward mechanisms and perceived social costs. This suggests that even small fluctuations in reward mechanisms and perceived social costs can lead to significant changes in online multimodal fake review generation. These two factors are the most critical in influencing online multimodal fake review generation. Additionally, variable with the largest negative expected impact is platform’s multimodal recognition and governance capabilities. This indicates that platform’s multimodal recognition and governance capabilities strongly inhibits multimodal fake review generation. Furthermore, findings remain consistent in male and female sample, where reward mechanisms and perceived social costs also have the highest positive expected impact on online multimodal fake review generation. Similarly, negative expected impact of multimodal recognition and governance capabilities is also the highest, emphasizing importance of enhancing platform’s capacity to govern fake reviews.

11 $$ Expected\,influence\,centrality\,value = \sum\limits_{u \in N(v)} {w_{uv} C(u)} $$

Note: $N(v)$ is set of nodes directly connected to node v, $w_{uv}$ is weight of edge between node u and node v, and $C(u)$ is some measure of centrality of node u, such as degree centrality, closeness centrality, or betweenness centrality.

A comprehensive comparison of centrality indicators suggests that, in full sample, closeness centrality and betweenness centrality exhibit similar trends, while strength centrality and expected influence centrality show roughly similar trends. Reward mechanisms indicators have relatively high values, indicating that this node plays a crucial role in online multimodal fake review generation. This may be attributed to users, driven by material rewards from merchants, being inclined to generate online multimodal fake reviews for benefits. Additionally, results of network centrality analysis exhibite heterogeneity between male and female samples, i.e., male sample has different trends in closeness centrality values and betweenness centrality values than female sample. Furthermore, when comparing results with importance ranking of feature factors obtained through machine learning technique, reward mechanisms are key feature factor of online multimodal fake review generation. This finding further validates robustness of our results.

## Discussion

In present study targeting China’s E-commerce platforms, determinants of online multimodal fake review generation are studied. Results show that determinants influencing online multimodal fake review generation are complex and interconnected. This study expands outcomes in previous research to some extent, especially concerning causal mechanisms of online multimodal fake review generation.

First, we find that platforms’ multimodal recognition and governance capabilities have a significant moderating effect on merchants’ fake review generation, but not on that of users. Results highlight influence of platforms’ signal governance on both merchant and user-generated fake review signals^206^. Empirical findings are consistent with previous research, providing support for proposed hypothesis^207–209^. We attribute this finding to our consideration of heterogeneous multi-agent complex signal interactions^210^. This suggests that platforms’ multimodal recognition and governance capabilities to regulate fake reviews can greatly impact merchants while having minimal influence on users. Studies indicate that platforms can effectively regulate merchants’ tendencies to induce online reviews, thereby limiting impact of merchant-generated reviews^211–213^. Consequently, adoption of specific review-generating regulatory strategies represents good option for platforms, elucidating stringent measures implemented by certain e-commerce platforms against merchant-generated reviews, such as Taobao’s limitation of rewarding positive reviews and offering cashback promotions^214–216^. Specifically, the stronger platforms’ multimodal recognition and governance capabilities, the greater limiting effect on merchants’ online multimodal fake review generation. However, impact of user-generated online multimodal fake reviews remains unaffected by platforms’ multimodal recognition and governance capabilities^217,218^. This result may be attributed to fact that users do not bear any costs for generating fake reviews, whereas merchants bear some social costs for generating fake reviews^219^. Additionally, Binder et al.^220^ investigate into influence of platform regulation on volume of online reviews generated by merchants and consumers, highlighting greater impact on merchants. Li et al.^221^ demonstrate that platforms’ signals for governing fake reviews significantly impact merchants’ online fake review generation but have no effect on consumers. Handan‐Nader et al.^222^ observe that platforms’ review governing signals exert stronger influence on merchants than on consumers in context of fake review generation. Dai et al.^223^ conclude that consumers are minimally impacted by platforms’ regulatory strategy and regulatory strength when generating product reviews.

Second, by introducing perceptual behavior control and quantifying its importance, this study demonstrates positive mediating role of perceptual behavior control in users’ multimodal fake review generation. This indicates that perceptual behavior control is an important determinant influencing users’ online multimodal fake review generation^224–226^. Our empirical evidence reveals that perceptual behavior control exerts both direct and indirect positive influence on users’ multimodal fake review generation. This indicates that users are external determinants in online multimodal fake review generation^227–229^. Specifically, within context of e-commerce platforms and driven by factors like emotional venting, perceived value, reward mechanisms, or subjective norms, users generate fake reviews through perceptual behavior control^230–232^. These findings not only complement but also extend outcomes found in partial mediation models proposed by Román et al.^233^, Petrescu et al.^234^, and Shahraki-Mohammadi et al.^235^. Furthermore, these findings are consistent with previous research, for example, Palese et al.^236^ demonstrate significant influence of consumers’ emotional attitudes and subjective norms on perceptual behavior control, which mediates interplay between emotional attitudes, subjective norms, and online review behaviors. Niechwiej-Szwedo et al.^237^ investigate into interrelation among positive Internet Word-of-Mouth (IWOM), perceptual behavior control in green consumption, and green consumption intention, revealing that positive IWOM significantly impacts both perceptual behavior control in green consumption and green consumption intentions, with the latter fully mediated by the former. In addition, Laszlo et al.^238^ explore determinants of users’ online knowledge payment behavior, highlighting partial mediation role of user-perceived behavior control in translating normative beliefs into intentions to pay for online knowledge services. Knijnenburg et al.^239^ reveal significant influence of consumers’ subjective norms and perceived behavior control on purchase intentions, with the latter serving as complete mediator between subjective norms and consumers’ purchase intentions.

Third, results show that reward mechanisms and perceived social costs emerge as the two most critical feature factors, reward mechanisms have the greatest impact on online multimodal fake review generation. Specifically, individuals are motivated to generate online product fake reviews to get various rewards, such as cash, points, or gift vouchers from merchants^240,241^. Outcomes of this investigation are consistent with frequently observed occurrences of positive reviews being exchanged for incentives. Moreover, these findings are consistent with results in existing literature, with many studies indicating significant positive impact of reward mechanisms on generating fake reviews^242,243^. While previous research points out that rewarding users is a key reason for disinformation generation^244^, they do not quantify degree of influence exerted by reward mechanisms. We find that reward mechanisms are primary determinants influencing users’ motivation to generate online multimodal fake reviews and quantify importance of reward mechanisms. This further enriches previous study^245,246^. Moreover, we highlight importance of perceived social costs, ranking it second in importance. This finding aligns with application of perceived cost theory to domain of online disinformation research^247^. Merchants perceive significant social pressure when interacting with other heterogeneous agents, thus driving them to be risk-averse^248^. Moreover, some researchers provide elucidation on this matter. For example, Chang et al.^249^ identify that perceived social costs significantly shape consumers’ inclination to share content within their social network on digital platforms. In addition, we find that merchants’ perceived psychological benefits do not affect online multimodal fake review generation, further substantiating previous structural equation modeling analyses. For such issue, Guan et al.^250^ reveal that online merchants who generate multimodal fake reviews demonstrate higher degree of risk aversion, as they carefully weigh costs associated with generating fake reviews. Importance of user-level motivational determinants is greater than that of merchant-level motivational determinants, likely due to the minimal cost users incur, as opposed to costs borne by merchants^251^. Furthermore, according to previous research^252,253^, we divide feature factors into distinct regimes based on their importance. Specifically, regime 1 encompasses reward mechanisms and perceived social costs. Regime 2 includes subjective norms, supporting/disparaging products, hiring review control agency, and merchant perception impact. Regime 3 includes supporting/disparaging merchants, perceptual behavior control, emotional venting, multimodal recognition and governance capabilities. Regime 4 encompasses perceived psychological benefits, user perception impact, and perceived value. This reasonable division of determinants is good for accurately identifying key determinants, thereby governing fake review generation. These findings significantly broaden existing research conducted by Shih et al.^28^ and Triberti et al.^29^ regarding determinants impacting fake review generation.

Finally, based on the study of Yang et al.^254^ and Kudo et al.^255^, this study analyzes intrinsic associations of determinants that affect online multimodal fake review generation in full sample and analyzes heterogeneity of male and female samples. Both in full sample, male sample, and female sample, reward mechanisms have the most significant influence on online multimodal fake review generation. This result indicates high possibility for both male and female consumers to generate online fake reviews to get material rewards from merchants. The finding is consistent not only with empirical observations but also with prior research in relative fields. For example, Deng et al.^256^ observe a similar phenomenon in their investigation of impact of leader’s reward neglect on employees’ propensity for silence. Additionally, perceived value, hiring review control agency, multimodal recognition and governance capabilities are strongly correlated, indicating a close interconnection between them. This implies that platforms’ multimodal recognition and governance capabilities are closely related to merchants’ online fake review generation behavior, a phenomenon explained by Sheng et al.^207^ and Ma et al.^208^. However, results of network centrality analysis also exhibite heterogeneity between male and female samples, i.e., male sample has different trends in closeness centrality values and betweenness centrality values than female sample. Effective interventions targeting these variables can yield significant results in terms of multimodal disinformation governance. This indicates that gender may play an important role in online fake review generation, leading to observable disparities in engagement of fake review generation between male and female consumers. Existing literature supports this opinion, for example, Fjendbo et al.^257^ explore impact of incentive performance pay on teachers’ motivation, and reveal that male teachers demonstrate greater emphasis on significance of performance-based compensation, and male teachers’ motivation appears to be more responsive to incentive performance pay in comparison to female teachers. Guenther et al.^258^ highlight dissimilarities of males and females responding to incentives when engaging in risky behaviors.

## Conclusions and future research

The majority of extant literature focuses on examining determinants, identification, and impact of fake review generation, but lacks insight into underlying causal mechanisms behind online fake review generation. Furthermore, prevalent scholarly works tend to rely on singular theoretical frameworks in investigating fake review generation, constructing research models that overlook influence of individual and mass interactions within the context of coexisting platforms, merchants, and users. Consequently, identified gaps in current research underscore imperative and significance of this study. The model we propose for fake review generation encompasses interactions among platforms, merchants, and users, offering valuable addition to current models investigating fake review generation. This research integrates the theories of signaling, actor-network, motivation, and human–environment interaction hypothesis to develop an original model elucidating mechanism of online multimodal fake review generation. The model takes into account individual and mass interactions in presence of multi-agents, including platforms, merchants, and users. Our study employs structural equation modeling to analyze online multimodal false review generation using data from China’s three leading e-commerce platforms, namely Taobao, Jingdong, and Pinduoduo. Additionally, our research investigates key determinants influencing online multimodal fake review generation through machine learning technique. By employing structural equation modeling and machine learning technique, our study uncovers causal mechanisms of online multimodal fake review generation and identifies reward mechanisms as key determinant influencing online multimodal fake review generation. Furthermore, our study reveals significant correlations among determinants contributing to online multimodal fake review generation.

This study presents several key theoretical implications. First, integration of the theories of signaling, actor-network, motivation, and human–environment interaction hypothesis serves as foundational framework, significantly broadening applicability of these established research paradigms. Second, by examining dynamics of individual and mass interactions among platforms, merchants, and users in shaping proliferation of online multimodal fake reviews, an original model is developed to elucidate generation mechanism under coalescence of these key agents. This model extends boundaries of information interaction theory and mechanisms for understanding fake review generation. Third, employing structural equation modeling, machine learning technique, and Bayesian complex networks, this study advances examination of online multimodal fake review generation, offering a fresh perspective on study of online fake reviews.

Considering bad impacts of fake reviews, our findings carry significant practical implications for merchants, online platforms, and public policy. First, merchants are advised to acknowledge that product quality, brand recognition, and authentic reviews serve as primary influences within digital marketplace. Rather than wasting resources towards fake review generation, efforts should be put towards enhancing brand integrity and fostering consumer confidence. Second, fake reviews can negatively affect both consumers and merchants, they also pose threats to online platforms. Investment in technological solutions, such as application of deep learning and other AI methods for bias management, review management, and service functionalities, is necessary. Third, online fake review necessitates attention from public policymakers. Guidelines should be implemented to curtail merchant manipulation through formulation of policies and provision of technical support, thereby ensuring sustained and healthy progression of e-commerce.

Despite these contributions, this study has several limitations, which serve to inform future research directions. First, environmental uncertainty may influence platforms’ multimodal recognition and governance capabilities, which we do not consider in this study. Hence, future researchers could explore impacts of environmental factors on online multimodal fake review generation. Second, incorporating individual differences and cross-cultural variables may uncover currently undiscussed variances. Subsequent studies could introduce moderating factors to expand the model. Finally, since this study may limit its generalizability as it is analyzed through a sample of platforms in China, more studies should be conducted in other countries and regions.

## Data Availability

Datasets used and/or analyzed during current study are available from corresponding author upon reasonable request.
