# Author Correction: Determinants of multimodal fake review generation in China’s E-commerce platforms

**DOI:** 10.1038/s41598-024-63720-6

**Published:** 2024-06-05

**Authors:** Chunnian Liu, Xutao He, Lan Yi

**Affiliations:** 1 School of Public Policy and Administration, Nanchang University, Nanchang, 330031 China; 2 Digital Literacy and Skills Enhancement Research Center, Jiangxi Province Philosophy and Social Science Key Research Base, Nanchang University, Nanchang, 330031 China

Correction to: *Scientific Reports* 10.1038/s41598-024-59236-8, published online 12 April 2024

The original version of this Article contained an error in Reference 26, which was incorrectly given as:

Badi, I., McCarty, D. & Kim, H. W. Risky behaviors and road safety: An exploration of age and gender influences on road accident rates. PLoS ONE 10.1371/journal.pone.0296663 (2024).

The correct reference is listed below:

McCarty, D. & Kim, H. W. Risky behaviors and road safety: An exploration of age and gender influences on road accident rates. PLoS ONE 10.1371/journal.pone.0296663 (2024).

The original Article has been corrected.

